# Response to first‐line treatment predicts progression‐free survival benefit of small‐cell lung cancer patients treated with anlotinib

**DOI:** 10.1002/cam4.3941

**Published:** 2021-05-06

**Authors:** Boyu Qin, Lingli Xin, Qingxiang Hou, Bo Yang, Jing Zhang, Xiaoguang Qi, Yingtian Wei, Yi Hu, Qi Xiong

**Affiliations:** ^1^ Department of Oncology General Hospital of Chinese PLA Beijing China; ^2^ Department of Gynaecology and Obstetrics PLA Rocket Force Characteristic Medical Center Beijing China; ^3^ Department of Radiology The First Medical Center of Chinese PLA General Hospital Beijing China

**Keywords:** anlotinib, efficacy, independent risk factor, small‐cell lung cancer

## Abstract

**Background:**

Anlotinib significantly extended progression‐free survival (PFS) and overall survival (OS) in small‐cell lung cancer (SCLC) as third or later line treatment.

**Methods:**

In this study, we retrospectively analyzed the efficacy and safety of anlotinib in the clinical practice and aimed to identify risk factors for predicting the clinical benefit of anlotinib in SCLC patients. 29 SCLC patients treated with anlotinib monotherapy or combination therapy as second or later line treatment were included. PFS, OS, objective response rate (ORR), disease control rate (DCR), and adverse events (AEs) were analyzed.

**Results:**

In whole patients, the median PFS was 2.1 months (95% confidence interval (CI): 1.1–3.2 months); The ORR and DCR were 10.3% and 48.3%, respectively; The median OS was 7.2 months (95%CI: 3.2–11.2 months). Cox regression analysis demonstrated that response to first‐line treatment was the independent risk factor for PFS. The ORR (20.0% vs. 0%) and DCR (53.3% vs. 42.9%) were promoted in patients treated with anlotinib combination therapy comparing to anlotinib monotherapy. The most common AEs were hoarseness, fatigue, decreased appetite, oral mucositis, and anemia. No treatment‐related AEs graded 3 or more.

**Conclusion:**

Anlotinib is an effective option for SCLC patients with tolerable toxicity as second or later line treatment. Patients sensitive to first‐line treatment had longer PFS when treated with anlotinib. Anloitnib combined with other therapy increased the efficacy without adding toxicity.

## Graphical abstract

In this study, we reported that anlotinib is an effective option for SCLC patients with tolerable toxicity as second or later line treatment. Patients sensitive to first line treatment gained more PFS benefit from anlotinib. Moreover, addition of anloitnib to chemotherapy or immunotherapy enhanced the efficacy in SCLC.

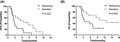

## 1 INTRODUCTION

Small‐cell lung cancer (SCLC) is a highly aggressive lung cancer with less than 1 year of median overall survival (OS) and lower than 5% of 5‐year survival rate.^1^, ^2^ Although SCLC patients respond well to the standard first‐line platinum‐based doublet chemotherapy, the majority of them experience relapsed disease. Recently, the addition of anti‐programmed death ligand‐1 (PD‐L1) antibodies to the first‐line chemotherapy prolonged about 3‐months longer median OS in extensive stage SCLC patients.^3^, ^4^ However, the disease in a great number of patients still progresses at 1 year after treatment. Unfortunately, multiple clinical trials of novel treatments, including anti‐PD‐1 antibodies and anti‐DLL3 antibody,^5^, ^6^, ^7^ fail to improve outcomes. Thus, there is a limited effective treatment option for relapsed SCLC patients.

Sufficient nutrition and oxygen supply for tumor progression need neo‐vascularization and neo‐angiogenesis. Anti‐angiogenesis therapy has been reported to promote efficacy in a variety of cancers. Nevertheless, previous studies suggested that bevacizumab (an antibody targeting vascular endothelial growth factor) combined with first‐line or second‐line chemotherapy did not improve OS in SCLC.^8^, ^9^, ^10^, ^11^ Other anti‐angiogenic therapies, including apatinib, sorafenib, vandetanib, and thalidomide, also failed to prolong progression‐free survival (PFS) or OS whether alone or combined with chemotherapy and introduced a high rate of toxicity.^12^, ^13^, ^14^ Surprisingly, only anlotinib significantly extended PFS and OS in the ALTER1202 study.

Anlotinib is a novel tyrosine kinase inhibitor (TKI) targeting receptor tyrosine kinases vascular endothelial growth factor receptor 1–3, epidermal growth factor receptor, fibroblast growth factor receptor 1–4, platelet‐derived growth factor receptor α and β, and stem cell factor receptor.^15^, ^16^, ^17^ The phase II clinical trial ALTER1202 showed that relapsed SCLC patients treated with anlotinib after two lines of chemotherapy had a significant prolonged PFS (4.1 months vs. 0.7 months) and OS (7.3 months vs. 4.9 months) than those treated with placebo.^18^ Based on these findings, anlotinib has been approved as third‐line treatment for SCLC by the China National Medical Products Administration in 2019. Another phase II study showed that the median PFS was 4.1 months and OS was 6.1 months in relapsed SCLC patients receiving anlotinib. Moreover, they indicated that limited‐stage patients had a longer OS than that of extensive‐stage patients.^19^ However, Chen et al. reported only 2.6 months of PFS in SCLC patients treated with anlotinib in the real‐world without reporting data of OS and AEs.^20^ Zhang et al. reported an advanced SCLC patient treated with anlotinib experienced 11 months duration of response after four lines of treatment.^21^ Considering the heterogeneity between patients included in clinical trial and real‐world and of different centers, we retrospectively investigated the efficacy and safety of anlotinib as second or later line treatment in SCLC patients in our center, and aimed to identify risk factors for predicting the clinical benefit of anlotinib.

## 2 METHODS

### 2.1 Patients and treatments

Pathologically confirmed SCLC patients receiving anlotinib as second or later line treatment in General Hospital of Chinese PLA between March 2019 and July 2020 were eligible for retrospective analysis. This study was approved by local ethics committee and conducted according to the principles of the Declaration of Helsinki. Considering the retrospective nature of this study, the requirement for informed consent was waived. The medical data, including sex, age, stage, presence of liver/brain metastasis, Eastern Cooperative Oncology Group Performance Status (ECOG PS), smoking history, previous treatment lines and regimens, response to first‐line treatment, history of radiotherapy, radiologic and laboratory data, were all collected.

Anlotinib was administered once daily (12 mg or 8 mg) for 14 days and discontinued for 7 days in one cycle. The starting dose of anlotinib was determined by the oncologist according to the patients’ status. The dosage of combined therapy was set according to the National Comprehensive Cancer Network or Chinese Society of Clinical Oncology guidelines. Follow‐up data were collected up to October 31st, 2020.

### 2.2 Assessments

Therapeutic effect was assessed according to Response Evaluation Criteria in Solid Tumors (RESICT) version 1.1 by radiologic data of computed tomography scans or magnetic resonance images by two doctors independently. The effect was categorized as complete response (CR), partial response (PR), stable disease (SD), or progressive disease (PD). When there was disagreement on radiologic evaluation, a third doctor was requested to reevaluate, and the agreement was reached by discussion. Patients who experienced PD in less than 3 months after first‐line treatment were considered refractory; patients progressed greater than 3 months after first‐line treatment were considered sensitive. The duration time from anlotinib administration to disease progression or death of any cause before disease progression was defined as PFS; while the time from the beginning of anlotinib administration to death was defined as OS. The rate of CR and PR was used to calculate ORR, and the rate of CR, PR, and SD was calculated as DCR. Treatment‐related AEs were graded using Common Terminology Criteria for Adverse Events version 5.0.

### 2.3 Statistical analysis

Continuous variables were described as median and 95% CI or range. Categorical variables were reported as frequency or percentage. Chi‐square test and Fisher's exact test were used to compare the difference between the two groups. ANOVA was performed to compare the difference among the three groups. Survival curves for PFS and OS were analyzed using the Kaplan–Meier method. The log‐rank test was used for univariate analysis between groups. Cox regression analysis was used to analyze the statistically significant risk factors according to results of univariate analysis. The risk factors with *p *< 0.2 in univariate analysis were considered significant and imported into Cox regression analysis. Statistical analysis was conducted by PRISM version 7.0 (GraphPad Software) and SPSS version 20.0 (IBM Corp.). Statistical significance was defined as *p *< 0.05 (two‐sided).

## 3 RESULTS

### 3.1 Baseline clinical characteristics of included patients

A total of 29 patients were eligible for analysis. Of the whole patients, 26 (89.7%) were male and 3 (10.3%) were female. The median age was 60 years old (range: 35–83). 24 (82.8%) patients were diagnosed with the extensive‐stage disease, while 5 (17.2%) patients were diagnosed with limited‐stage disease. 6 (20.7%) patients had liver metastasis and 14 (48.3%) patients had brain metastasis. There were 14 patients received anlotinib monotherapy and 15 patients who received anlotinib combination therapy. Of the 15 patients receiving combination therapy, 10 received anlotinib plus chemotherapy, 2 received anlotinib plus anti‐PD‐1/PD‐L1 therapy, 2 received anlotinib combined with anti‐PD‐1 antibody and chemotherapy, and 1 received anlotinib combined with anti‐angiogenesis and chemotherapy. 15 patients had received 1 line of previous therapy, and 14 patients received 2 or more lines of previous therapy. 16 patients were sensitive to the first‐line treatment, while 13 were refractory to first‐line treatment. The median follow‐up time was 5.0 months (mean: 7.1 months, range: 1.7–18.3 months). Other clinical characteristics were summarized in Table 1.

**TABLE 1:** Demographics and baseline characteristics of patients included

Characteristics	Total (*n* = 29)	Monotherapy (*n* = 14)	Combination therapy (*n* = 15)	*p* value
Sex				1.000
Male	26 (89.7)	13 (92.9)	13 (86.7)	
Female	3 (10.3)	1 (7.1)	2 (13.3)	
Age				0.109
≤65	21 (72.4)	8 (57.1)	13 (86.7)	
>65	8 (27.6)	6 (42.9)	2 (13.3)	
Stage				0.651
Limited‐Stage	5 (17.2)	3 (21.4)	2 (13.3)	
Extensive‐Stage	24 (82.8)	11 (78.6)	13 (86.7)	
Liver metastases				0.169
No	23 (79.3)	13 (92.9)	10 (66.7)	
Yes	6 (20.7)	1 (7.1)	5 (33.3)	
Brain metastases				0.466
No	15 (51.7)	6 (42.9)	9 (60.0)	
Yes	14 (48.3)	8 (57.1)	6 (40.0)	
Smoking history				0.093
Never smoked	5 (17.2)	2 (14.3)	3 (20)	
Current smoker	3 (10.3)	0 (0)	3 (20)	
Former smoker	21 (72.4)	12 (85.7)	9 (60)	
ECOG PS				0.100
≤1	26 (89.7)	11 (78.6)	15 (100)	
>1	3 (10.3)	3 (21.4)	0 (0)	
No. of previous treatment lines				1.000
<2	15 (51.7)	7 (50.0)	8 (53.3)	
≥2	14 (48.3)	7 (50.0)	7 (46.7)	
Previous radiotherapy				1.000
No	10 (34.5)	5 (35.7)	5 (33.3)	
Yes	19 (65.5)	9 (64.3)	10 (66.7)	
Response to first‐line treatment				0.066
Refractory	13 (44.8)	9 (64.3)	4 (26.7)	
Sensitive	16 (55.2)	5 (35.7)	11 (73.3)	

Data were present as *n* (%) unless specified. The *p* value was for comparison between monotherapy and combination therapy.

### 3.2 Clinical efficacy of anlotinib in whole patients

**FIGURE 1:**
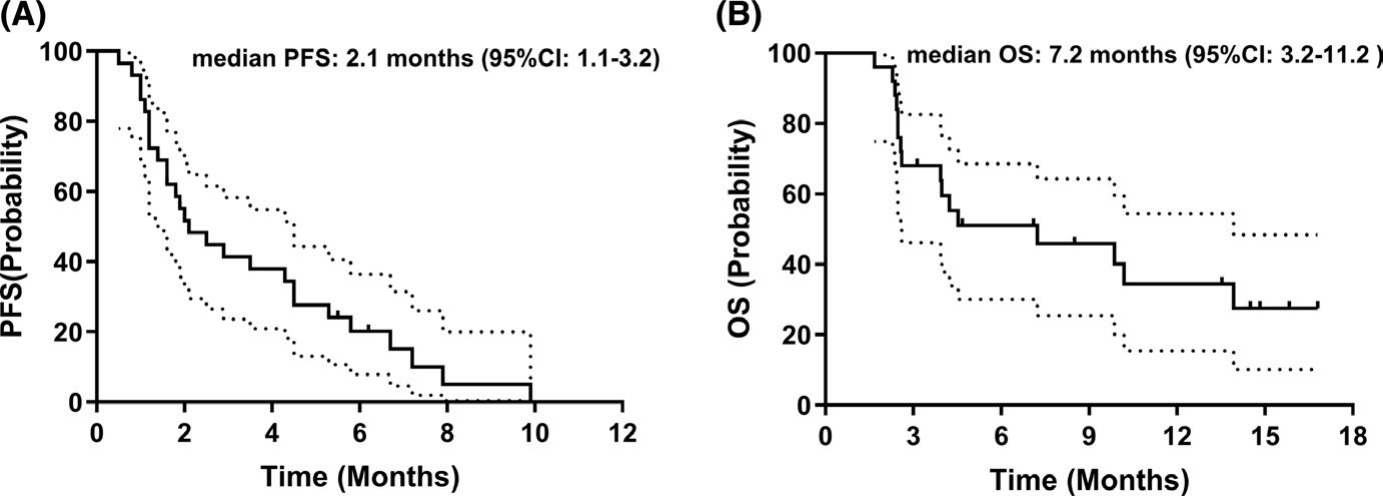
The PFS and OS for all included patients

**FIGURE 2:**
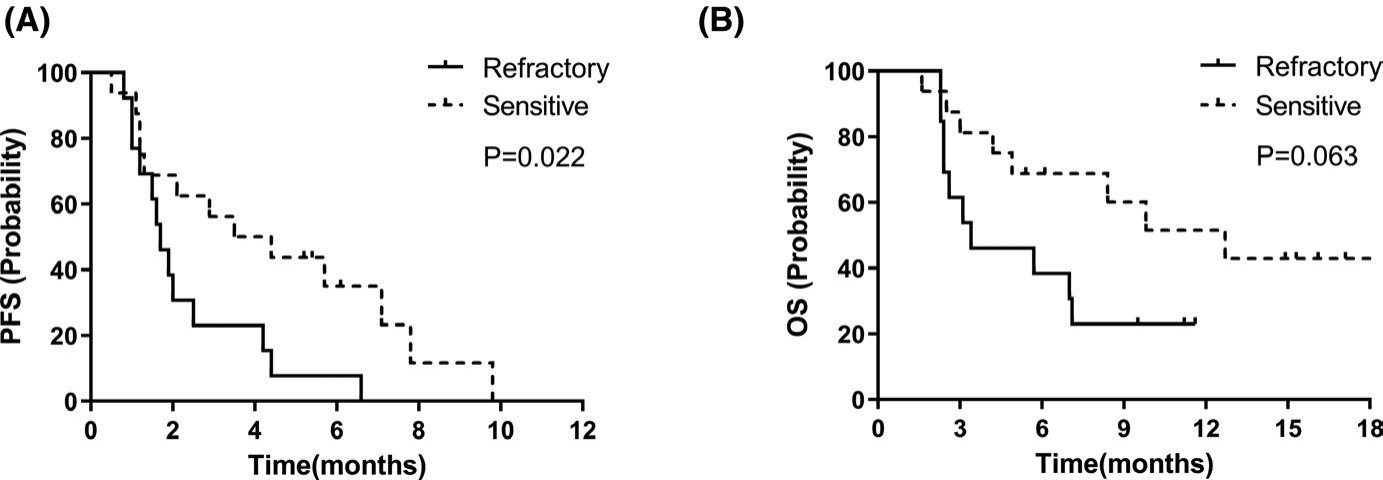
Comparison of PFS and OS between refractory patients and sensitive patients

Of the 29 patients, 3 were assessed as PR, 11 were SD, and 15 were PD according to the evaluation criteria of RESICT 1.1. The ORR and DCR were 10.3% and 48.3%, respectively. The overall median PFS was 2.1 months (95%CI: 1.1–3.2 months) (Figure 1A). Univariate analysis showed that patients with age <65 years old, brain metastases, smoking, sensitive to first‐line treatment had prolonged PFS; while sex, stage, liver metastases, ECOG PS, lines of previous treatment, previous radiotherapy, the therapeutic strategy had no influence on PFS (Table 2). Subsequent Cox regression analysis demonstrated that response to first‐line treatment was the only independent risk factor for PFS (Figure 2, Table 3).

**TABLE 2:** Univariate analysis of progression‐free survival and overall survival

Characteristics	PFS		OS	
	mPFS (95%CI)	*p* value	mOS (95%CI)	*P* value
Sex		0.249		0.258
Male	1.9 (1.275–2.525)		5.7 (1.446–9.954)	
Female	6.6 (3.989–7.611)		8.4 (6.833–12.701)	
Age		0.105		0.013
≤65	3.5 (0.360–6.640)		12.7 (8.227–14.359)	
>65	1.7 (1.284–2.116)		3.1 (0.000–7.396)	
Stage		0.353		0.887
Limited‐Stage	1.5 (1.071–1.929)		2.6 (2.171–3.029)	
Extensive‐Stage	2.5 (0.580–4.420)		7.1 (3.493–10.707)	
Liver metastases		0.662		0.976
No	2.1 (1.161–3.039)		7.0 (4.004–9.996)	
Yes	1.2 (0.000–4.201)		9.8 (0.000–24.304)	
Brain metastases		0.152		0.673
No	1.7 (1.069–2.331)		5.7 (0.000–13.555)	
Yes	2.5 (1.033–3.967)		7.1 (4.965–9.235)	
Smoking history		0.162		0.164
Never smoked	4.2 (2.697–5.703)		14.3 (9.336–19.224)	
Current smoker	9.8 (0.010–13.590)		10.7 (3.979–17.421)	
Former smoker	1.9 (1.302–2.498)		5.7 (1.740–9.660)	
ECOG PS		0.435		0.234
≤1	2.1 (0.000–4.474)		8.4 (1.769–15.031)	
>1	2 (1.840–2.160)		7.0 (0.000–14.362)	
No. of previous treatment lines		0.335		0.066
<2	2.1 (1.343–2.857)		7 (2.253–11.747)	
≥2	1.7 (0.000–5.183)		11.6 (7.758–15.585)	
Previous radiotherapy		0.245		0.144
No	1.6 (1.290–1.910)		3.1 (0.000–7.370)	
Yes	2.9 (0.909–4.891)		8.4 (5.795–11.005)	
Response to first‐line treatment		0.022		0.063
Refractory	1.7 (1.23–2.17)		3.4 (0.000–7.040)	
Sensitive	3.5 (0.56–6.44)		12.7 (5.924–19.476)	
Combination therapy		0.446		0.176
No	1.9 (1.350–2.45)		4.9 (0.683–9.117)	
Yes	3.5 (0.000–7.161)		12.7 (6.096–19.304)	

**TABLE 3:** Cox regression analysis of progression‐free survival and overall survival

Risk Factor	PFS		OS	
	HR (95%CI)	*p* value	HR (95%CI)	*p* value
Age	0.858 (0.273–2.701)	0.794	0.980 (0.245–3.924)	0.978
Brain metastases	0.560 (0.224–1.404)	0.216		
Smoking history				
Never smoked		0.294		0.472
Current smoker	0.706 (0.063–7.900)	0.777	4.468 (0.202–98.911)	0.343
Former smoker	2.189 (0.616–7.772)	0.226	4.234 (0.415–43.233)	0.223
No. of previous treatment lines			0.520 (0.164–1.655)	0.268
Previous radiotherapy			0.547 (0.180–1.665)	0.288
Response to first‐line treatment	0.318 (0.110–0.921)	0.035	0.425 (0.122–1.481)	0.179
Combination therapy			0.581 (0.154–2.196)	0.423

The overall median OS was 7.2 months (95%CI: 3.2–11.2 months) (Figure 1B). Univariate analysis showed that age, smoking status, previous treatment lines, previous radiotherapy, response to first‐line treatment and therapeutic strategy were risk factors related to OS (Table 2). However, these factors demonstrated no significant influence on OS in Cox regression analysis (Figure 2, Table 3).

### 3.3 Comparison of efficacy between anlotinib monotherapy and combination therapy

**FIGURE 3:**
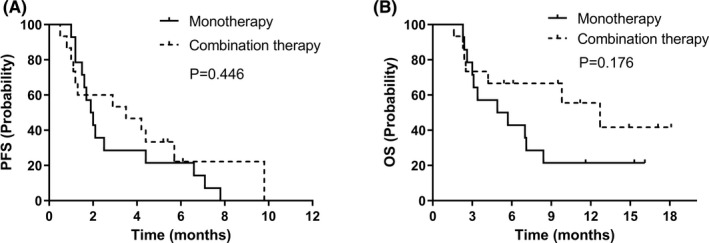
Comparison of PFS and OS between patients treated with anlotinib monotherapy and combination therapy

There were 14 patients in anlotinib monotherapy group and 15 patients in anlotinib combination therapy group. We found that patients receiving anlotinib combination therapy had longer PFS and OS (median PFS: 3.5 vs. 1.9 months; median OS: 12.7 vs. 4.9 months) than those treated with anlotnib monotherapy, though the difference was not significant (Figure 3). Our results also showed that the 6‐month and 12‐month survival rates were higher in patients treated with anlotinib combination therapy (Table 4). The ORR and DCR were 20.0% and 53.3% in patients of anlotinib combination therapy group comparing to 0% and 42.9% in patients of anlotinib monotherapy group. No significant difference between ORR and DCR were found.

**TABLE 4:** Overall response and survival of anlotinib monotherapy and combination therapy

	Monotherapy	combination therapy	*p* value
CR	0	0	
PR	0	3	
SD	6	5	
PD	8	7	
ORR (%)	0 (0.0)	3 (20.0)	0.224
DCR (%)	6 (42.9)	8 (53.3)	0.715
PFS			
Events, *n* (%)	14 (100.0)	12 (80.0)	
Median, months, (95%CI)	1.9 (1.350–2.450)	3.5 (0.000–7.161)	
6‐month rate, % (95%CI)	14.3 (0.0–32.7)	22.2 (0.0–46.1)	
12‐month rate, % (95%CI)	—	—	
OS			
Events, *n* (%)	11 (78.6)	7 (46.7)	
Median, months (95%CI)	4.9 (0.683–9.117)	12.7 (6.096–19.304)	
6‐month rate, % (95%CI)	42.9 (17.0–68.8)	66.7 (42.8–90.6)	
12‐month rate, % (95%CI)	21.4 (0.0–43.0)	55.6 (27.6–83.6)	

### 3.4 Safety analysis

AEs were reported in 27 (93.1%) patients. On the whole, all AEs were grade 1–3 whether in patients receiving anlotinib monotherapy or combination therapy, and no discontinuation of anlotinib and treatment‐related death was observed. The five most common AEs were hoarseness (37.9%), fatigue (37.9%), decreased appetite (37.9%), oral mucositis (27.6%) and anemia (27.6%). The reported grade 3 AEs were hoarseness (6.9%), fatigue (3.4%), anemia (3.4%), hand‐foot syndrome (3.4%) and hypokalemia (6.9%). In patients treated with anlotinib monotherapy, the five most common AEs were fatigue, hoarseness, decreased appetite, anemia, oral mucositis; while in patients treated with anlotinib combination therapy, the five most common AEs were decreased appetite, hoarseness, fatigue, rash, and leucopenia. Six patients reported mild bleeding and managed well with the administration of hemostatic agents. All reported AEs were summarized in Table 5.

**TABLE 5:** Safety analysis

Adverse event	All Patients (*n* = 29)	Monotherapy (*n* = 14)		Combination therapy (*n* = 15)		
	Any grade	≥3 grade	Any grade	≥3 grade	Any grade	≥3 grade
Hoarseness	11 (37.9)	2 (6.9)	5 (35.7)	1 (7.1)	6 (40)	1 (6.7)
Fatigue	11 (37.9)	1 (3.4)	6 (42.9)	1 (7.1)	5 (33.3)	0 (0)
Decreased appetite	11 (37.9)	0 (0)	5 (35.7)	0 (0)	6 (40)	0 (0)
Anemia	8 (27.6)	1 (3.4)	4 (28.6)	0 (0)	4 (26.7)	1 (6.7)
Oral mucositis	8 (27.6)	0 (0)	4 (28.6)	0 (0)	4 (26.7)	0 (0)
Leucopenia	6 (20.7)	0 (0)	1 (7.1)	0 (0)	5 (33.3)	0 (0)
Hand‐foot syndrome	6 (20.7)	1 (3.4)	2 (14.3)	1 (7.1)	4 (26.7)	0 (0)
Hemoptysis	6 (20.7)	0 (0)	2 (14.3)	0 (0)	4 (26.7)	0 (0)
Increased AST	5 (17.2)	0 (0)	2 (14.3)	0 (0)	3 (20)	0 (0)
Rash	5 (17.2)	0 (0)	0 (0)	0 (0)	5 (33.3)	0 (0)
Nausea	5 (17.2)	0 (0)	4 (28.6)	0 (0)	1 (6.7)	0 (0)
Thrombocytopenia	4 (13.8)	0 (0)	3 (21.4)	0 (0)	1 (6.7)	0 (0)
Diarrhea	4 (13.8)	0 (0)	4 (28.6)	0 (0)	0 (0)	0 (0)
Hypertension	2 (6.9)	0 (0)	0 (0)	0 (0)	2 (13.3)	0 (0)
Increased ALT	2 (6.9)	0 (0)	0 (0)	0 (0)	2 (13.3)	0 (0)
Hypokalemia	2 (6.9)	2 (6.9)	1 (7.1)	1 (7.1)	1 (6.7)	1 (6.7)
Constipation	2 (6.9)	0 (0)	1 (7.1)	0 (0)	1 (6.7)	0 (0)
Increased Scr	1 (3.4)	0 (0)	1 (7.1)	0 (0)	0 (0)	0 (0)

Abbreviations: ALT, alanine transaminase; AST, aspartate transaminase; Scr, serum creatinine.

## 4 DISCUSSION

Relapsed SCLC patients currently have limited effective treatments. In the ALTER 1202 study, the SCLC patients receiving anlotinib as a third or later line treatment had a significant improved survival with 4.1 months PFS and 7.3 months OS.^18^ Wu et al. reported that the median PFS was 4.1 months and the median OS was 6.1 months in relapsed SCLC patients treated with anlotinib.^19^ The ORR of the previous two studies was approximate 5%. Comparing to results of these two clinical trials, our retrospective study showed that the median PFS (2.1 months) was poorer, which was similar to another retrospective study (median PFS: 2.6 months)^20^; while the median OS (7.2 months) was similar, and ORR was higher (10.3%). The different clinical benefit of anlotinib between our study and others might lie in the following reasons: the ALTER 1202 study and the phase II study by Wu et al. were both prospective and only enrolled patients treated with anlotinib monotherapy as third or later line treatment, while our study was retrospective and enrolled patients receiving anlotinib monotherapy and anlotinib plus other therapy as second or later line treatment, which probably attributed to the higher ORR in our study; the rate of brain metastasis (48.3%) was much higher than those in previous studies, and we speculated that this might partially explain the poor PFS in our study, since SCLC patients with brain metastasis have worse prognosis.^22^ Collectively, ours and previous studies suggested that anlotinib was an effective drug for relapsed SCLC patients, considering there was limited option for those patients.

The benefit from later line treatments depends on the response to initial first‐line treatment, according to which patients were categorized as sensitive or resistant relapse.^23^ In present study, our results suggested that response to first‐line treatment was an independent prognostic risk factor for PFS of SCLC patients treated with anlotinib. Patients who were sensitive to first‐line treatment gained more PFS benefit from anlotinib. Whereas, it was not an independent risk factor for OS. But we did observe a tendency of longer OS in patients responding well to first‐line treatment. Future study with a bigger sample size is needed to confirm our findings. In the phase II study by Wu et al., they indicated that the OS for SCLC patients of limited‐stage disease treated with anlotinib was significantly prolonged^19^; while in the ALTER1202 study, OS was prolonged in patients with brain metastasis.^18^ However, our results showed that the clinical stage and brain metastasis were not independent risk factors for PFS and OS in patients treated with anlotinib. The difference may be due to the small sample size and different therapeutic strategy among the studies.

Previous studies suggested that anti‐angiogenesis therapy combined with other type of therapies had synergistic effects on NSCLC. Han et al. reported that anlotinib combined with chemotherapy or target therapy increased ORR and DCR in NSCLC patients.^24^, ^25^ Here, we compared the efficacy of anlotinib monotherapy and combination therapy in SCLC patients. We found that the ORR (20% vs. 0) and DCR (53.3% vs. 42.9%) were promoted in patients receiving anlotinib combination therapy. We also found that the PFS and OS were marginally prolonged. Due to the small sample size, analysis of risk factors could not be performed in this study. However, our study supported the notion that anlotinib combination therapy could enhance the efficacy. A future study with bigger sample size is needed to investigate prognostic risk factors for SCLC patients treated with anlotinib.

Concerning the AEs, we did not observe new AEs compared with previous studies focusing on anlotinib in SCLC patients. We found that hoarseness (37.9%), fatigue (37.9%), decreased appetite (37.9%) were the most common AEs whether in anlotinib monotherapy or combination therapy. In this study, no grade 3–5 hypertension, which is the most frequent AE in patients receiving anlotinib, was reported and the incidence of hypertension was relatively lower than the previous study.^19^ We pondered this might due to the following reasons: firstly, the previous study was prospective, while ours was retrospective; secondly, the sample size in our study was relatively small; thirdly, the dosage of anlotinib was set at 12 mg initially in other studies, but the initial dosage was 8 mg or 12 mg in our study. Fatal bleeding was the most concerned AE in patients treated with anti‐angiogenesis. There were 6 patients reporting mild bleeding but no life‐threatening bleeding in our study. All the 6 patients continued anlotinib after oral administration of hemostatic agents. Here, we also showed that anloitnib combination therapy increased the efficacy without adding new AEs. Thus, our results showed that anlotinib is well tolerable in SCLC whether alone or combined with other therapy.

Some limitations of this study should be noted. The major concern is the relatively small sample size of our retrospective, single‐center study, which leads to a few subgroups without available data and lowers the statistical power. Large scale prospective study is needed to confirm our results. In addition, the positive results of anlotinib in SCLC might be due to larger number of targets of anlotnib than other anti‐angiogenic TKIs.^26^ Though no validated biomarker has been identified for antiangiogenic drugs, study on NSCLC indicated that genetic alternations of *ARID1A*, *BRCA2* and *IDH* ^exon4^ could potentially be used to guide anlotinib therapy.^27^ Thus, analysis of gene status would play a pivotal role in identifying patients likely to benefit from anlotinib. This was not performed in our study and was another limitation.

In conclusion, anlotinib is an effective option for SCLC patients with tolerable AEs as second or later line treatment. We found that patients sensitive to first‐line treatment had longer PFS when treated with anlotinib. Moreover, we showed that anlotinib in combination with other therapy prolonged PFS in patients with limited‐stage disease and OS in patients with liver metastases.

## CONFLICT OF INTEREST

The authors declare that there is no conflict of interest.

## Data Availability

All Data available on request from the authors.
